# T Cells, Triglycerides, and the Immune Roots of Residual Cardiovascular Risk

**DOI:** 10.1016/j.jacbts.2025.101376

**Published:** 2025-09-22

**Authors:** Michael D. Shapiro

**Affiliations:** Center for Prevention of Cardiovascular Disease, Department of Cardiovascular Medicine, Wake Forest University School of Medicine, Winston-Salem, North Carolina, USA

**Keywords:** adaptive immunity, interleukin-6, lipid immunobiology, residual cardiovascular risk, residual inflammatory risk, triglyceride-rich lipoproteins

**Figure:**
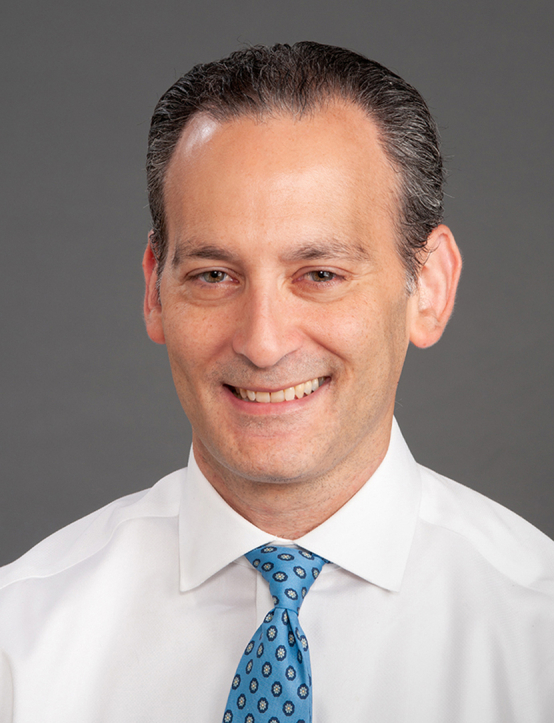

For decades, elevated triglycerides (TGs) have hovered at the periphery of cardiovascular risk assessment: measurable, modifiable, yet maddeningly elusive in causal interpretation. Whereas observational studies and Mendelian randomization analyses have repeatedly pointed to moderate hypertriglyceridemia as a causal contributor to atherosclerotic cardiovascular disease (ASCVD),^1^^,^^2^ therapeutic trials targeting TGs have returned mostly disappointing results.^3^^,^^4^ Reilly et al^5^ offer a new mechanistic perspective in this issue of *JACC: Basic to Translational Science*, suggesting that TGs may exert their atherogenic effects not simply through lipotoxicity or remnant accumulation, but via direct modulation of adaptive immune cell phenotypes.

Their work represents a methodologically elegant and biologically provocative analysis of circulating T cells from individuals with varying degrees and etiologies of hyper- and hypotriglyceridemia. The principal finding, an inflammatory transcriptomic signature in CD4^+^ and CD8^+^ T cells from individuals with moderate primary hypertriglyceridemia, raises the possibility that triglyceride-rich lipoproteins (TRLs) may reprogram lymphocyte function in ways that promote vascular inflammation. Among the differentially expressed genes in this subgroup, the up-regulation of the interleukin-6 receptor (IL6R) is particularly notable given IL-6’s well-established role as a causal effector of ASCVD.^6^ This finding aligns with and extends recent translational evidence implicating immune cell modulation in the residual inflammatory risk observed in statin-treated populations.^7^

Importantly, this study arrives at a critical juncture. The concept of “residual risk” has become a default explanation for persistent events despite low-density lipoprotein cholesterol lowering, yet its mechanistic underpinnings remain poorly defined. By identifying a proinflammatory immune phenotype linked to moderate hypertriglyceridemia, distinct from either normotriglyceridemia or severe hypertriglyceridemia, this work sheds light on a candidate pathway that may bridge the gap between lipoprotein physiology and vascular inflammation. It challenges the orthodoxy that TGs are secondary actors in atherogenesis and reframes them as active participants in immune cross talk.

The investigators employed bulk RNA-sequencing on highly purified CD4^+^ and CD8^+^ T cells from 5 well-defined patient groups: control, primary and secondary moderate hypertriglyceridemia, primary severe hypertriglyceridemia (familial chylomicronemia syndrome), and hypotriglyceridemia (hypobetalipoproteinemia). The most robust transcriptomic activation, particularly involving inflammatory and IL-6–related pathways, was observed in primary moderate hypertriglyceridemia. Interestingly, this signal was reversed in patients with hypotriglyceridemia and absent in severe hypertriglyceridemia, where TGs are largely sequestered in chylomicrons too large to penetrate the endothelium.

There are 3 core contributions of this study. First, it challenges the prevailing reductionist assumption that lipoproteins are only passive vehicles for lipid deposition. Instead, it supports a growing paradigm in which lipoproteins, particularly TRLs, may serve as bioactive immunomodulators. Second, it suggests that not all hypertriglyceridemia is biologically equivalent. The absence of an inflammatory T-cell signature in severe hypertriglyceridemia, despite markedly elevated TGs, reinforces the view that particle size, lipolytic remnants, and cell access matter more than total concentration. Third, the study identifies IL6R as a potential interface between lipid metabolism and immune activation, reinforcing the therapeutic rationale for IL-6 pathway inhibition in ASCVD.

The IL-6 axis has emerged as one of the few inflammation-related pathways with both genetic and pharmacologic validation in atherosclerosis. Mendelian randomization studies have shown that lifelong reduction in IL-6 signaling is associated with lower ASCVD risk, and the CANTOS (The Canakinumab Anti-inflammatory Thrombosis Outcome Study]) trial definitively demonstrated that IL-1β inhibition (an upstream activator of IL-6) reduces cardiovascular events.^8^ The T-cell–specific up-regulation of IL6R expression observed in moderate hypertriglyceridemia in this study may represent a plausible cellular mechanism linking lipid exposure to inflammatory risk. This also raises the question of whether IL-6 signaling within lymphocytes, rather than monocytes or hepatocytes, is a relevant therapeutic target.

This study also echoes observations from parallel domains. For example, oxidized phospholipids carried on lipoprotein(a) have been shown to drive T-cell activation and vascular inflammation.^9^ The concept of lipoproteins as immune signaling platforms is supported by converging evidence. TRLs may now be added to the growing list of lipoprotein species that function as immunologic cofactors in atherogenesis.

However, before rushing to redraw our understanding of TG biology, several limitations warrant mention. The most obvious is sample size: only 49 individuals were included, with as few as 3 or 4 participants in the severe and hypotriglyceridemia groups. This inherently limits generalizability and statistical power for subgroup comparisons. Moreover, the design is cross-sectional, precluding any inference of causality or directionality. The observed transcriptomic signatures may reflect chronic immune reprogramming from TG exposure, or they may be epiphenomena secondary to unmeasured confounding.

Reilly et al^5^ attempted to address confounding by conducting extensive sensitivity analyses, including adjustments for cardiovascular history, statin or ezetimibe use, PCSK9 inhibitors, and apolipoprotein B levels. Even though these corrections strengthened confidence in the observed associations, they cannot fully rule out residual confounding from diet, physical activity, microbiome composition, or unmeasured inflammatory comorbidities. Furthermore, the functional implications of the observed transcriptional changes remain unclear. An up-regulation of IL6R at the messenger RNA level is intriguing, but whether this translates into enhanced signaling, protein expression, or altered effector function of T cells remains unknown.

One might also question whether the focus on T cells is too narrow. Monocytes and macrophages remain the canonical immune effectors in atherosclerosis, and the role of T cells, particularly CD8^+^ subsets, is still evolving. Whereas prior studies have shown that fatty acids can modulate T-cell metabolism and function,^10^ it is not yet clear how these transcriptomic findings integrate with established models of plaque formation and rupture. A further level of complexity involves T-cell plasticity and lineage commitment. Whether TG-rich environments skew naive T cells toward specific proatherogenic phenotypes (eg, Th1, Th17) or impair regulatory T-cell function remains an open question.

Nonetheless, this study is both timely and important. The failure of the PROMINENT (Triglyceride Lowering with Pemafibrate to Reduce Cardiovascular Risk) trial to reduce ASCVD events despite lowering TGs with pemafibrate has cast renewed doubt on the TG hypothesis. But such outcomes may reflect a mismatch between the therapy and the target. Perhaps lowering TGs is not enough if the inflammatory sequelae of TRLs persist.^3^ The work by Reilly et al^5^ suggests that immune reprogramming, rather than lipid clearance alone, may be a more proximal and tractable target.

From a clinical perspective, these findings should encourage a reappraisal of the patient with moderate hypertriglyceridemia. Rather than viewing TGs as a metabolic nuisance or a cosmetic lipid abnormality, clinicians may need to consider whether elevated TGs serve as a flag for immune activation, and whether such patients merit not only lipid-lowering therapy but potentially anti-inflammatory interventions as well. TRLs do not circulate in isolation. They are a product of, and a marker for, broader metabolic dysfunction. The inflammatory T-cell phenotype observed here may be as much a reflection of insulin resistance, hepatic steatosis, or visceral adiposity as of circulating TGs per se. Thus, interventions aimed at correcting the underlying metabolic terrain through dietary quality, physical activity, and weight loss may be equally or more important than lipid-targeted or cytokine-based therapies.

More provocatively, these data raise the possibility that moderate TG elevations might serve as a biomarker not only of metabolic risk, but of immune activation. If validated in larger cohorts, T-cell transcriptomic profiling could potentially stratify patients with hypertriglyceridemia into distinct inflammatory phenotypes. This could open the door to precision prevention strategies, in which lipid-lowering and anti-inflammatory therapies are tailored based on immune readouts rather than conventional lipid panels alone.

Where do we go from here? Several avenues are worth pursuing. First, functional assays of T-cell cytokine production, activation, and proliferation in response to TRLs should be conducted in vitro and in animal models. Second, larger and longitudinal human studies should assess whether T-cell transcriptomic signatures predict clinical ASCVD events independently of TG levels. Third, interventional studies using anti-inflammatory agents, particularly IL-6 antagonists, could explore whether patients with moderate hypertriglyceridemia and a proinflammatory T-cell profile derive particular benefit. Fourth, combining transcriptomic profiling with proteomics, lipidomics, and single-cell sequencing may reveal cellular pathways that are currently hidden in bulk RNA data.

Lastly, these findings should prompt investigators to revisit prior null results through a more mechanistic lens. Trials like PROMINENT were not designed to select patients based on inflammatory endophenotypes. The same may be true for other populations with elevated TGs but differing immune states, such as those with HIV, chronic kidney disease, or autoimmune conditions. The next generation of TG-focused research should not simply ask whether lowering TGs works, but in whom, and under what immunometabolic conditions.

In cardiovascular disease prevention, we are quick to quantify what we can measure and slow to investigate what we cannot. Reilly et al^5^ remind us that residual risk is not a number, it is a process. And understanding that process means embracing the immune system not just as a bystander, but as an active accomplice in lipid-mediated disease.

The path from TGs to atherothrombosis may be paved, at least in part, by T cells.

## Funding Support and Author Disclosures

Dr Shapiro has received grant/research support (through his institution) from Amgen, Arrowhead, Boehringer Ingelheim, Esperion, Novartis, Ionis, Merck, New Amsterdam, Lilly, and Cleerly; has participated in Scientific Advisory Boards with Amgen, Arrowhead, Esperion, Ionis, Novartis, New Amsterdam, and Merck; and has served as a consultant for Ionis, Novartis, Regeneron, Novo Nordisk, Arrowhead, and Tourmaline.
